# Hemodynamics and renal function during administration of low-dose dopamine in severely ill patients

**DOI:** 10.1590/S1516-31802004000400002

**Published:** 2004-07-01

**Authors:** Cláudia Nathalie Pereira, Flávia Ribeiro Machado, Hélio Penna Guimarães, Ana Paula Resque Senna, José Luiz Gomes do Amaral

**Keywords:** Dopamine, Hemodynamic processes, Intensive care units, Diuresis, Diuretics, Dopamina, Processos hemodinâmicos, Unidades de Terapia Intensiva, Diurese, Diuréticos

## Abstract

**CONTEXT:**

Although a large number of studies have been performed regarding the renal and hemodynamic effects of the infusion of low-dose dopamine (LDD) in severely ill patients, there is still controversy on this subject.

**OBJECTIVE:**

To evaluate the effects of dopamine (2 μg/kg/min) on systemic hemodynamics (lowest mean arterial pressure, MAP, highest heart rate, HR, central venous pressure, CVP), creatinine clearance (CLcr), diuresis and fractional sodium excretion (FENa^+^).

**TYPE OF STUDY:**

A non-randomized, open, prospective clinical trial.

**SETTING:**

An intensive care unit in a tertiary university hospital.

**PARTICIPANTS:**

22 patients with hemodynamic stability admitted to the intensive care unit.

**PROCEDURES:**

Patients were submitted to three two-hour periods: without dopamine (P1), with dopamine (P2) and without dopamine (P3).

**MAIN MEASUREMENTS:**

The abovementioned variables were measured during each period. CLcr was assessed based upon the formula U x V/P, where U is urinary creatinine (mg/dl), V is diuresis in ml/min and P is serum creatinine (mg/dl). FENa was calculated based upon the formula: urinary sodium (mEq/l) x P/plasma sodium (mEq/l) x U) x 100. Results were presented as mean and standard deviation. The Student t test was used and results were considered significant if p was less than 0.05.

**RESULTS:**

Twelve patients (seven males and five females) were included, with a mean age of 55.45 years. There was no significant variation in MAP, HR, CVP, CLcr or FENa with a dopa-mine dose of 2 μg/kg/min. On the other hand, diuresis significantly increased during P2, from 225.4 to 333.9 ml.

**CONCLUSION:**

Infusion of 2 μg/kg/min of dopa-mine for 2 hours increases diuresis. At the doses studied, dopamine does not induce significant alterations in MAP, HR, CVP, CLcr and FENa^+^.

## RESUMO

**CONTEXTO:**

Apesar do grande número de trabalhos sobre os efeitos renais e hemodinâmicos de doses baixas de dopamina em pacientes graves, ainda existe muita controvérsia a respeito.

**OBJETIVOS:**

Esse estudo objetivou avaliar os efeitos de dopamina (2 mcg/kg/min) na hemodinâmica (máxima pressão arterial média, PAM, freqüência cardíaca máxima, FR, pressão venosa central, PVC, *clearance* de creatinina, CLcr, diurese e fração de excreção de sódio, FENa^+^).

**TIPO DE ESTUDO:**

Estudo clínico não randomizado, aberto, prospectivo.

**LOCAL:**

Uma unidade de terapia intensiva em hospital universitário terciário.

**PARTICIPANTES:**

22 pacientes com estabilidade hemodinâmica admitidos na Unidade.

**PROCEDIMENTOS:**

Os pacientes foram submetidos a três períodos de duas horas de duração cada um: sem dopamina (P1), com dopamina (P2) e sem dopamina (P3).

**PRINCIPAIS VARIÁVEIS ESTUDADAS:**

As variáveis acima mencionadas foram medidas em cada um dos períodos. CLcr foi medido baseado na fórmula U.V/P, onde U é a creatinina urinária (mg/dl), V é a diurese em ml/min e P é a creatinina sérica (mg/dl). FENa^+^ foi calculado baseado na fórmula: sódio urinário (mEq/l) x P/sódio plasmático (mEq/l) x U) x 100. Os resultados foram apresentados como média e desvio padrão. O teste t de Student foi utilizado e os resultados considerados significativos se o valor de p fosse menor que 0,05.

**RESULTADOS:**

12 pacientes (sete homens e cinco mulheres) foram incluídos, com a idade média de 55.45 anos. Não houve nenhuma variação na PAM, FR, PVC, CLcr ou FENa^+^ com o uso de dopamina na dose de 2 mcg/kg/min. Entretanto, a diurese aumentou significativamente no P2, de 225.4 para 333.9 ml.

**CONCLUSÕES:**

A infusão de 2 mcg/kg/min de dopamina durante duas horas aumenta a diurese. Nas doses estudadas, a dopamina não induziu alterações significativas na PAM, FR, PVC, CLcr e FENa^+^.

## INTRODUCTION

Dopamine is an endogenous catecholamine, and is the immediate metabolite of norepinephrine and epinephrine. At low concentrations of 0.2 to 5 μg/kg/min, dopamine acts on two populations of dopaminergic receptors (DA), called the DA1 and DA2 receptors. DA1 receptors are post-synaptic and are located in the smooth muscle cells of renal, mesenteric and coronary gastric vessels and in hepatic arteries.^1^ DA2 receptors are pre-synaptic and are located in the adrenal medulla and sympathetic nerve endings.^1^ Binding of dopamine to these two specific receptors causes arterial vasodilation,^2^ as well as increased glomerular filtration, renal blood flow and fractional sodium excretion.^3,4^

Acute renal failure is relatively frequent among severely ill patients. Although the introduction of biocompatible membranes into hemodialysis has contributed towards increasing the survival rate of acute renal failure patients, this condition is associated with poor prognosis, especially when associated with multiple organ dysfunction.^2^

The term “dopaminergic effect” is used to refer to stimulus of the dopaminergic receptors (and possibly the beta receptor), without changes in the arterial pressure.^5^ This effect is achieved with dopamine doses of less than 3 μg/kg/min (called “dopaminergic doses”). Many believe that the infusion of 2 μg/kg/min of dopamine may benefit renal function, thereby increasing electrolyte excretion and urine output.^5-7^ Such opinions have led to the routine use of low-dose dopamine (LDD) in situations with the risk of renal failure. Indeed, because of its hemodynamic and renal effects, LDD has been used in severely ill patients in an effort to minimize renal dysfunction.

Notwithstanding this recommendation, doubts remain concerning the beneficial effects of this procedure.^3,6,8-11^ Studies with animals^12^ and acute renal failure patients^8,9,13-17^ have not shown evidence that LDD maintained or improved renal function.

Thus, if proven inadequate, continued injection of LDD would result only in potential additional medical treatment,^18^ thereby increasing the costs. The objective of the present study was to evaluate the effect of the continuous injection of 2 μg/kg/min of dopa-mine on the systemic hemodynamics and renal function of patients under intensive care.

## MATERIAL AND METHODS

This study was a non-randomized, open, prospective clinical trial, conducted within a surgical intensive care unit of a tertiary university hospital: Hospital São Paulo, Universidade Federal de São Paulo – Escola Paulista de Medicina (Unifesp – EPM). Following approval of the research protocol by the hospital's Ethics Committee, 22 patients admitted to the intensive care unit were included in the trial after giving their informed consent. The inclusion criteria were that they should be aged over 18 and present hemodynamic stability. Patients were excluded if they were currently using vaso-active drugs or had previously been treated with dopamine, if they presented coronary disease or acute/chronic renal failure, and if there was no indication for bladder catheterization.

The patients included were classified according to the Acute Physiology and Chronic Health Evaluation II (APACHE II) disease severity system.^19^ For the duration of the study, the crystalloid infusion and ventilation support remained unchanged and no new drugs were administered.

Patients were evaluated over a six-hour period divided into three periods of two hours. In the first period (P1), saline solution was infused at a volume corresponding to what would be needed for infusing 2 μg/kg/min of dopamine. In the second period (P2), 2 μg/kg/min of dopamine were administered. In the third period (P3), saline infusion was resumed.

At the end of each period, diuresis (UV) was established and a sample for urinary sodium (UNa^+^-mmol/l) and urinary creatinine (UCr, μmol/l) assaying was collected. At these times, blood samples were also collected to assess plasma sodium (PNa^+^, mmol/l) and plasma creatinine (PCr, μmol/l) levels. From the data obtained above, the creatinine clearance (CLcr = UCr/PCr x UV) and the fractional sodium urinary excretion (FENa^+^ = UNa^+^ x PCr/PNa^+^ x UCr) were calculated. During these periods, the hemodynamic values, heart rate (HR), mean arterial pressure (MAP) and central venous pressure (CVP) were measured every 30 minutes.

The results are presented as mean and standard deviation. For statistical analysis, the Student t test was used, with p < 0.05 as the significance level.

## RESULTS

A total of 22 adult patients of both genders (9 men and 13 women) were studied, with a mean age of 55.45 years (Table 1). The APACHE score results and diagnoses upon admission to the intensive care unit are presented in Tables 2 and 3, respectively. The data collected and presented in Tables 4 and 5 show that there were no significant variations in HR, MAP and CVP during the three periods studied.

**Table 1:** Anthropometric data of 22 patients admitted in a surgical intensive care unit in São Paulo

Variable	Value
Age (years)	55.45 ± 22.69 (16-89)
Weight (kg)	69.48 ± 12.58 (50-95)
Height (cm)	1.68 ± 0.08 (1.53-1.80)
Gender (male/female)	9/13

*Results are expressed in mean ± standard deviation (range).*

**Table 2:** Classification of the disease severity (according to Acute Physiology and Chronic Heath Evaluation II, APACHE II) of 22 patients admitted in a surgical intensive care unit in São Paulo

APACHE II	Patients
< 1*5*	4
15-25	13
> 25	5

**Table 3:** Individual diagnosis upon admission at intensive care unit of 22 patients in São Paulo

Patients	Diagnosis
AWG	Esophagus carcinoma (PO)
LTC	Politraumatism
NSA	Politraumatism
AO	Status epilepticus
JRP	Retroperitoneal abcess (PO)
EMG	Meningioma (PO)
ONO	Medular compression (PO)
AR	Acute pancreatitis
AV	Squamous cell carcinoma (PO perfusion)
SBR	Femur fracture (PO)
EM	Medular compression (PO)
JG	Aortic aneurism (PO)
WO	Myasthenia gravis
ECS	Pulmonary metastatic cancer (PO)
MJO	Cerebral aneurism (PO)
AGA	Extradural hematoma (PO)
LAP	Hernia hiatal (PO)
JB	Pneumonia
RZM	Ectopic pregnancy (PO)
GCA	Esophagus carcinoma (PO)
FCB	Medular compression (PO)
MHA	Rip fracture (PO)

*PO = admission at immediate postoperative period.*

**Table 4:** Individual values for hemodynamic parameters in the three phases (P1, P2, and P3) of the study with 22 patients admitted in an intensive care unit in São Paulo

Patients	Mean arterial pressure (mmHg)			Heart rate (bpm)			Central venous pressure (mmHg)		
	P1	P2	P3	P1	P2	P3	P1	P2	P3
AWG	110	100	97	94	121	110	14.0	10.0	12.0
LTC	70	80	70	109	105	114	14.0	15.0	14.0
NSA	75	95	90	77	87	87	11.0	11.0	11.0
AO	70	80	75	81	93	77	15.0	12.0	13.0
JRP	60	70	60	118	122	119	8.0	11.0	14.0
EMG	85	85	80	103	102	101	-	-	-
ONO	90	90	95	74	73	79	-	-	-
AR	110	110	100	95	85	76	8.0	8.0	8.0
AV	92	104	116	99	90	90	11.0	11.0	11.0
SBR	100	112	111	81	82	108	8.5	9.0	9.0
EM	97	99	96	88	96	90	9.0	10.0	10.0
JG	90	87	80	94	98	95	14.0	13.0	15.0
WO	87	90	80	105	100	103	11.0	10.0	11.0
ECS	106	106	100	100	93	97	10.0	15.0	15.0
MJO	96	90	77	120	120	115	16.0	14.0	15.0
AGA	86	87	91	86	87	91	12.0	12.0	12.5
LAP	93	87	97	105	102	104	12.0	11.0	11.5
JB	110	100	110	91	93	92	13.0	14.0	13.0
RZM	95	97	110	115	135	140	17.0	17.5	15.0
GCA	95	110	90	85	92	105	15.0	13.0	15.0
FCB	85	95	90	96	100	95	15.0	13.0	13.5
MHA	76	73	80	77	87	84	17.0	17.5	17.5

**Table 5:** Hemodynamic analysis of 22 patients admitted in an intensive care unit in São Paulo during the three phases of the study (P1, P2, and P3)

Variable	P1	P2	P3
Heart rate (bpm)	95.1 ± 13.43	98.3 ± 14.86	98.7 ± 15.33
Arterial pressure (mmHg)	89.9 ± 13.63	93.0 ± 11.66	90.7 ± 14.41
Central venous pressure (mmHg)	12.5 ± 2.91	12.4 ± 2.60	12.8 ± 2.38

*Results are expressed in mean ± standard deviation.*

**Figure 1:**
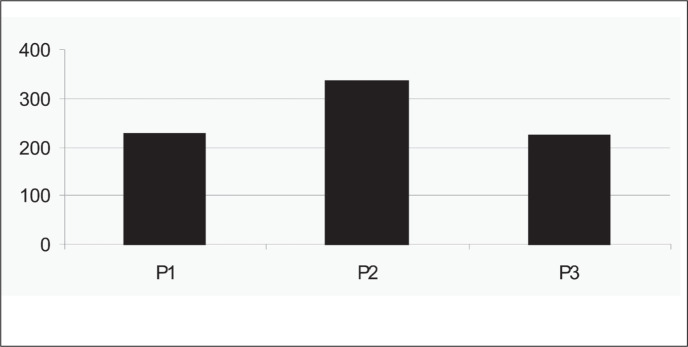
Diuresis (in ml) during the three infusion periods (P1, P2, and P3) in 22 patients admitted in na intensive care unit in São Paulo. Results are expressed in means.

The individual values for serum creatinine, creatinine clearance, fractional sodium excretion and diuresis for the three periods are presented in Table 6. During the P2 period, during LDD infusion, there was a significant increase in the diuresis volume, in comparison with the P1 and P3 periods (Table 7 and Figure 1). No significant variations in CLcr or FENa^+^ (Table 7) were recorded during the periods studied.

**Table 6:** Individual values for renal parameters in 22 patients admitted in an intensive care unit in São Paulo in the three phases of the study (P1, P2, and P3)

Patients	Serum creatinine (mg/dl)			Creatinine clearance (ml/min)			Fraction of sodium excretion			Diuresis (ml)		
	P1	P2	P3	P1	P2	P3	P1	P2	P3	P1	P2	P3
AWG	0.8	0.7	0.5	142.3	168.9	-	0.666	1.350	-	112	346	86
LTC	0.9	0.9	0.9	155.8	87.1	87.0	1.079	0.756	-	306	147	336
NSA	0.6	0.6	0.6	166.1	218.8	165.0	2.033	2.128	2.700	427	525	463
AO	0.6	0.5	0.6	118.8	129.1	297.0	0.142	0.829	0.132	186	352	320
JRP	1.1	1.1	1.2	139.1	298.1	63.0	0.656	0.853	1.376	102	305	114
EMG	0.9	0.9	0.8	172.5	147.5	74.0	1.259	1.387	1.451	207	175	217
ONO	1.0	0.9	1.0	69.6	162.7	171.0	1.620	0.996	2.593	522	676	542
AR	1.7	1.6	1.9	133.6	240.0	127.0	0.177	0.276	0.374	213	388	235
AV	1.3	1.2	1.2	208.0	250.0	179.0	0.500	0.300	0.700	200	250	220
SBR	0.7	0.5	0.6	171.0	135.0	54.0	-	0.900	3.000	70	200	210
EM	0.8	0.5	0.6	231.0	230.0	194.0	0.700	1.100	1.100	250	300	250
JG	1.0	1.2	1.2	116.5	67.2	49.0	0.919	0.759	0.901	190	220	100
WO	0.6	0.6	0.6	87.5	152.0	101.2	0.787	0.565	0.250	180	240	185
ECS	0.6	0.5	0.6	309.2	145.1	90.1	1.370	0.352	1.222	305	55	102
MJO	0.6	0.7	0.7	110.6	138.3	107.4	1.915	2.333	0.747	325	440	160
AGA	0.7	0.8	0.9	161.1	65.3	41.2	3.561	7.601	1.918	410	352	80
LAP	0.7	0.7	0.7	275.4	149.6	104.1	0.363	1.365	2.160	120	245	270
JB	0.8	0.9	0.9	51.6	85.6	115.9	1.526	1.691	1.538	110	560	400
RZM	0.6	0.6	05	129.2	197.2	96.3	0.818	1.024	0.587	150	355	105
GCA	1.1	0.8	0.9	75.0	134.4	63.9	0.278	0.812	0.451	132	645	115
FCB	0.7	0.7	0.6	141.7	65.0	232.5	0.763	0.542	0.393	350	455	310
MHA	0.8	0.7	0.8	108.3	116.4	108.9	0.233	0.184	0.225	92	115	95

**Table 7:** Renal function of 22 patients admitted in an intensive care unit in São Paulo during the three phases of the study (P1, P2, and P3)

Variable	P1	P2	P3	p
Diuresis (ml)	225.40 ± 122.70	333.90 ± 165.3	223.4 ± 129.4	< 0.05
Clearance of creatinine (ml/min)	148.80 ± 63.30	153.80 ± 63.60	120.10 ± 65.20	ns
Fraction of sodium excretion	1.0 ± 0.8	1.3 ± 1.5	1.2 ± 0.8	ns

*Results are expressed in mean ± standard deviation. ns = not significant.*

## DISCUSSION

Since dopamine was first synthesized in 1910, a large amount of research related to this drug has allowed identification of its effects and mechanism of action,^2,8,20^ and also definition of its therapeutic possibilities. The idea that LDD could prevent acute renal failure arose naturally from knowledge of its selective renal vasodilatory properties. Acute renal failure is characterized by increased plasma creatinine levels and decreased glomerular filtration rate. This syndrome has a high mortality rate, which justifies the interest in its prevention.

The hemodynamic effects from the infusion of increasing doses of dopamine have been studied by D'Orio et al. (1984).^20^ These authors found that the activity of this agent on β 1 receptors was reflected in improved cardiac output, while stimulation of α1 receptors led to increased systemic arterial pressure. They furthermore concluded that, at doses equal to or smaller than 2 μg/kg/min, dopamine has selective action on the dopaminergic receptors. Stimulation of β1 receptors can be perceived at doses as small as 3 μg/kg/min.

Among the patients of the present study, no significant variations in HR, CVP or MAP were observed, which is in agreement with various other studies. Indeed, LDD seems to have no cardiac effects. In patients at the postoperative stage of cardiac surgery, Sato et al. (1982)^21^ detected that LDD had a favorable effect on renal hemodynamics, without changing the cardiac output. Similar results were achieved by Lherm et al. (1996)^11^ in patients with septic syndrome and septic shock. Strigle & Petrinec (1990),^22^ in an experimental model, did not detect pressure changes in the systemic or pulmonary occluded arteries.

With regard to renal function, many studies undertaken among normal individuals or patients with different diagnoses have shown a beneficial effect. Some previous studies, and even more recent ones, have shown an improvement in renal function in a variety of settings, such as situations of high intra-abdominal pressure during laparoscopy,^23^ cardiac surgery,^24,25^ refractory ascites due to cirrhosis,^26^ liver transplantation,^27^ general intensive care^28,29^ or postoperative states. Some authors have even suggested that the responsiveness to LDD, as assessed by two-hour creatinine clearance, could be helpful in selecting patients who would benefit from its use.^30^ Although these studies have shown beneficial effects, most of them only included small numbers of patients and did not evaluate the long term effects of LDD. In a recent study, LDD was found to increase creatinine clearance, diuresis and fractional sodium excretion, without concomitant hemodynamic change. These effects reached a maximum during an eight-hour period of LDD infusion. But despite the persistence of a slight increase in diuresis, the improvement in creatinine clearance and FENa^+^ disappeared after 48 hours.^28^ According to these data, it is likely that tolerance to dopamine-receptor agonists develops in critically ill patients.

However, this beneficial effect has not been consistently confirmed by other authors. Even the short-term effects of LDD were not confirmed by the present study, in which no differences in creatinine clearance were found. Corroborating our results, other authors have also shown that renal function is not improved with LDD for patients undergoing cardiac surgery^16^ or liver transplantation,^17^ or for those in postoperative states,^15,16,31^ critically ill with oliguria,^13^ or with previous renal diseases.^14^ Other recent studies have confirmed these findings. In a double-blind, randomized, multicenter, placebo-controlled study with a large number of severely ill patients (n = 328),^18^ treatment with LDD did not seem to be advantageous. Patients with at least two systemic inflammatory response syndrome criteria and evidence of early renal dysfunction (oliguria or increased serum creatinine levels) did not present any protective effect, as assessed by peak creatinine levels. There was no difference in the evolution and severity of renal dysfunction, or in the length of hospital or intensive care unit stay.^18^ Although this is the largest study performed to assess this question, one possible bias to be taken into account is the lack of control over the volemic resuscitation rate. The possible effect of administering other drugs that could potentially affect renal perfusion or diuresis also needs to be considered.^18^ In a recently published meta-analysis including 970 patients, no improvement in serum creatinine levels or the incidence of renal function were detected.^32^ The same conclusion was reached in a recently published review on this subject.^33^

When analyzing the renal effects of LDD, a distinction must be made between increased diuresis and the biochemical improvement of renal function. It is important to take into account that there is no clearly direct relationship between these two variables, and that excessive diuresis may be harmful. With LDD infusion, the patients in the present study showed a significant increase in diuresis, of 48%, which corroborates the findings of other researchers.^12,17,27,34-37^ Although some of these authors argue that a significant increase in diuresis after LDD infusion could protected the kidney, for instance against the hazardous effects of cross-clamping of the aorta,^35^ there is no strong evidence that this really occurs. Nonetheless, despite the increased diuresis, our patients did not have a corresponding increase in CLcr. This therefore suggests the absence of a direct consistent relation between CLcr and diuresis. Thus, dopamine seems to have no beneficial effects and may increase diuresis, with the potential risk of jeopardizing the patient's volemic state. This is potentially deleterious in critically ill patients, for whom hypovolemic states must be avoided as a general rule. Moreover, among patients with mild to moderate renal failure or diabetes mellitus undergoing coronary angiography and receiving contrast, LDD seemed to have no advantage over volume replacement in preventing acute renal failure.^38^

It is therefore questionable whether renal function can be improved by the use of LDD, although increased diuresis is likely to occur, at least in some patients, as shown in our study. Thus, the question to be answered is whether it is better to use LDD or other diuretics when the objective is to increase diuresis. One potential benefit of LDD is its more continuous and homogeneous effect, which could be indicated in some clinical situations. However, although we could not demonstrate any cardiovascular effects from LDD, there is a potential concern about using a vasoactive drug instead of a diuretic when the desired effect is to increase urine output. In preeclampsia/eclampsia patients with persistent oliguria after adequate volume replacement, LDD was similar in effect to furosemide, in relation to diuresis improvement and the need for hemodialysis.^39^ Although there has not yet been any definitive answer to this question, it seems that LDD is not superior to diuretics.

Another aspect that needs to be assessed is the potential effect of LDD on sodium metabolism. The effect of LDD on natriuresis may be explained by its inhibition of aldosterone,^14^ and by its direct action on the proximal renal tubules.^10^ In some studies in which a significant increase in sodium excretion was detected, the patients were suffering from congestive cardiac failure,^4,12^ but other studies have found higher excretion even in patients without cardiac disease.^28,37^ It has been suggested that the action of aldosterone (commonly found at high rates in congestive cardiac failure) would be antagonized by the action of LDD on the DA2 receptors, thereby increasing diuresis and the output of Na^+^.^2^ This matter continues to be controversial, since other authors have failed to detect this effect.^4,9,17,40^ In the present investigation the increase in FENa^+^ (30%) was not considered significant.

## CONCLUSION

We have shown that the infusion of 2 μg/kg/min of dopamine for two hours increased the diuresis but did not changed CLcr and FENa^+^. This is in agreement with many other studies on humans that have not demonstrated the prevention of acute renal failure in high-risk patients or an improved outcome for those with established acute renal failure. We did not find any cardiovascular effects from LDD. However, while the safety profile of LDD in these settings has not been extensively defined, it is known that dopamine may precipitate serious cardiovascular and metabolic complications in critically ill patients. As the use of dopamine seems to have no beneficial effects and may increase diuresis, with a potential for compromising the patient's volemic state, its use with such indication ought to be avoided in intensive care unit patients. However, in hypervolemic patients, dopamine use may be considered. We suggest that LDD should not be used for selective renal vasodilatory and natriuretic actions in patients with acute renal failure until their efficacy has been established in randomized controlled trials. However, the use of dopamine for its systemic effects in heart failure and septic shock should not be precluded, since this may have beneficial effects.
